# 肺腺癌软脑膜转移患者的脑脊液和血液中不同的基因突变谱：基于循环肿瘤DNA的液体活检

**DOI:** 10.3779/j.issn.1009-3419.2020.102.14

**Published:** 2020-08-20

**Authors:** 会颖 李, 宇 谢, 永娟 林, 婷婷 俞, 震宇 尹

**Affiliations:** 210008 南京, 南京大学医学院附属鼓楼医院老年肿瘤科 Department of Geriatric Oncology, Affiliated Nanjing Drum Tower Hospital of Nanjing University Medical School, Nanjing 210008, China

**Keywords:** 肺肿瘤, 软脑膜转移, 脑脊液, 循环肿瘤DNA, 二代基因测序, Lung neoplasms, Leptomeningeal metastases, Cerebrospinal fluid, Circulating tumor DNA, Next generation sequencing

## Abstract

**背景与目的:**

软脑膜转移(leptomeningeal metastasis, LM)是晚期非小细胞肺癌(non-small cell lung cancer, NSCLC)的一种严重并发症, 通常预后较差。对于驱动基因阳性的NSCLC患者, 表皮生长因子受体酪氨酸激酶抑制剂(epidermal growth factor receptor-tyrosine kinase inhibitors, EGFR-TKIs)是治疗的首选, 但常伴随着无法解决的耐药问题。本研究旨在探讨NSCLC-LM患者的脑脊液(cerebrospinal fluid, CSF)和血液中不同的基因突变谱及突变丰度, 筛查相关耐药基因, 以期能精确指导个体化的治疗。

**方法:**

采用二代基因测序(next generation sequencing, NGS)的液体活检技术, 检测、对比分析TKI耐药后NSCLC患者同期的外周血及脑脊液中的循环肿瘤DNA(circulating tumor DNA, ctDNA)。

**结果:**

共纳入18例NSCLC伴有LM的患者, 基础突变中11例(61.11%)为*EGFR*, 6例(33.33%)为间变性淋巴瘤激酶(anaplastic lymphoma kinase, *ALK*), 1例(5.56%)为原癌基因酪氨酸蛋白激酶*ROS*(ROS proto-oncogene 1, receptor tyrosine kinase, *ROS1*), 伴随突变中肿瘤蛋白*P53*(tumor protein *p53* gene, *TP53*)、间质-上皮细胞转化因子(mesenchymal-epithelial transition factor, *MET*)检出率相对较高。CSF样本的靶向基因突变检出率明显高于外周血(100.00% *vs* 66.67%, *P*=0.006), 且基础突变基因的CSF丰度均明显高于血浆样本(*P* < 0.001)。CSF样本检测出丰富的单核苷酸变异(single-nucleotide variations, SNV)和拷贝数变异(copy number variants, CNV), 数量均多于血液样本；且相较于只接受过单一TKI治疗的患者, 使用多种TKI后脑脊液和血液中会产生更多的SNV突变。

**结论:**

NSCLC-LM患者CSF中的ctDNA, 相较于外周血, 能更加准确、全面地反映出LM的真实基因突变状态, 在指导用药、耐药监测、预后评估等方面具有广泛的应用前景, 可作为液体活检的优选。

## Abstract (en)

**Background and objective:**

Leptomeningeal metastasis (LM) are a severe complication of non-small cell lung cancer (NSCLC), and normally accompanied by poor prognosis. For the patients with targetable mutations, epidermal growth factor receptor-tyrosine kinase inhibitors (EGFR-TKIs) are the preferred treatment, but the acquired TKI resistance is inextricable. The aim of this study is to analyze the different gene mutation spectrum and mutation frequency of the cerebrospinal fluid (CSF) and plasma in NSCLC patients with LM, and screen out the drug-resistant mutations so as to guide the choice of treatment accurately.

**Methods:**

The paired CSF and plasma samples were collected from the NSCLC-LM patients with acquired TKI resistance. Next generation sequencing (NGS) was used to detect the gene variations of circulating tumor DNA (ctDNA).

**Results:**

A total of 18 NSCLC patients with LM were collected. Of the basic mutations, 11 cases (61.11%) were *EGFR*, 6 cases (33.33%) were anaplastic lymphoma kinase (*ALK*), and 1 case (5.56%) was *ROS* proto-oncogene 1, receptor tyrosine kinase (*ROS1*). Tumor protein *p53* gene (*TP53*) and mesenchymal-epithelial transition factor (*MET*) were the two most frequently accompanying mutated genes in CSF ctDNA. The detected mutation rate of CSF samples was higher than that of plasma samples (100.00% *vs* 66.67%, *P*=0.006), and the maximum allelic fractions were all higher in CSF than in plasma (*P* < 0.001). Abundant single-nucleotide variations (SNV) and copy number variants (CNV) were detected in CSF, the amount of both of which were more than in blood. In addition, the CSF and plasma samples of patients treated with several TKIs had more SNV mutations than patients who received only a single TKI treatment.

**Conclusion:**

For the patients of NSCLC, ctDNA in CSF could reveal genomic alterations of LM more exactly and overally than it in plasma, thus could be an optimal source of liquid biopsy for guiding therapy, monitoring therapeutic effect, and predicting prognosis.

软脑膜转移(leptomeningeal metastasis, LM)是晚期非小细胞肺癌(non-small cell lung cancer, NSCLC)的一种严重并发症, 发生率约为3%-5%^[1]^。而在表皮生长因子受体(epidermal growth factor receptor, *EGFR*)突变阳性的患者中, 由于EGFR-酪氨酸激酶抑制剂(EGFR-tyrosine kinase inhibitors, EGFR-TKIs)的广泛使用, 生存期得到明显的延长, LM的发生率可达到9.4%^[2]^。LM的治疗目前仍缺乏标准有效的方案, 患者的预后较差, 中位生存期仅为3个月-11个月左右^[3]^。

对于驱动基因阳性的NSCLC患者, TKIs仍然是治疗的首选, 可以带来明确的生存获益^[3]^, 但常伴随着无法解决的耐药问题。T790M是较为常见的耐药突变, 此外人类表皮生长因子受体2(human epidermal growth factor receptor 2, *erbB-2*)、间质-上皮细胞转化因子(mesenchymal-epithelial transition factor, *MET*)等基因表达的扩增, 肿瘤蛋白P53(tumor protein *p53* gene, *TP53*)、鼠类肉瘤病毒癌基因(kirsten rat sarcoma viral oncogene, *KRAS*)等基因的错义突变, 间变性淋巴瘤激酶(anaplastic lymphoma kinase, *ALK*)及肉瘤致癌因子-受体酪氨酸激酶1(*ROS* proto-oncogene 1, receptor tyrosine kinase 1, *ROS1*)融合基因等都会对TKI的疗效产生影响, 最终导致耐药从而病情进展^[4]^。因此在出现TKIs耐药后, 重新明确其转移灶的基因突变状态对于指导后续的治疗显得尤其重要。对于颅内转移灶, 组织活检由于标本获取困难及有创性, 临床应用受限。而基于二代基因测序(next generation sequencing, NGS)的液体活检技术, 为肺癌精准治疗提供了更加安全、有效的途径。液体活检可通过检测外周血、脑脊液(cerebrospinal ﬂuid, CSF)等体液中的循环肿瘤DNA(circulating tumor DNA, ctDNA), 判断NSCLC患者的肿瘤负荷、药物的疗效以及耐药情况^[5]^。此外有研究^[6, 7]^证实CSF中的ctDNA相较于外周血, 能更加准确地反映颅内转移瘤的基因突变情况。

基于此, 本研究通过ctDNA测序技术比较TKI耐药后NSCLC患者同期的脑脊液及外周血中的基因突变谱和突变频率, 筛查相关的耐药基因, 探讨耐药机制, 以期能预测疗效并指导后续个体化的治疗。

## 1 资料和方法

### 1.1 资料来源

本研究筛选了2013年1月-2019年10月期间就诊于南京鼓楼医院的经病理明确诊断的NSCLC患者, 其中42例经头颅磁共振成像(magnetic resonance imaging, MRI)或脑脊液细胞学明确诊断为软脑膜转移, 最终入组18例可获取同期的脑脊液及外周血样本的患者(详细筛选流程见图 1), 均已签署相关知情同意书。

**1:**
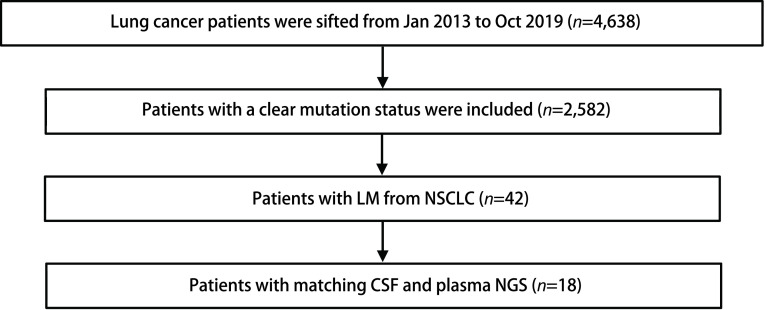
研究筛选流程图 Study flow chart. LM: leptomeningeal metastasis; NSCLC: non-small cell lung cancer; CSF: cerebrospinal fluid; NGS: next generation sequencing.

### 1.2 检测方法

患者入组后暂时停用TKIs, 且不接受化疗、放疗或免疫治疗。经腰椎穿刺留取脑脊液约10 mL, 抽取同期的外周血约8 mL于EDTA抗凝管中, 并在2 h内以2, 000 rpm离心10 min分离血浆, 然后统一保存于-80 ℃冰箱中。样本借助南京世和基因生物技术有限公司提供的Qiagen DNA提取试剂盒提取ctDNA, 超声波将其破碎为300 bp-350 bp。经96rxn xGen Exome Research Panel v1.0试剂盒富集后建立高通量测序文库, 再采用Illumina Hiseq高通量测序平台进行深度测序, 具体操作步骤参照试剂盒说明书进行。利用生物信息学平台整合分析下机数据, 最终生成样本的突变信息。检测共覆盖139个肺癌相关基因的外显子、融合相关内含子、可变剪切区域及特定微卫星位点区域等。质控环节包括样本制备、文库构建、靶向富集、样本上机、高通量测序、数据质控。要求样本至少应达到未损坏的DNA > 200 ng, ctDNA样本提取总量 > 50 ng, 如经质控分析显示损毁或不符合前述质控要求的样品将被退回。

### 1.3 统计学方法

采用SPSS 23.0软件进行统计学分析。实验结果应用单因素方差分析, *P* < 0.05为差异有统计学意义。

## 2 结果

### 2.1 基本情况

共纳入18例NSCLC伴有LM的患者, 年龄33岁-62岁, 中位年龄48岁, 男9例, 女9例, 男女比例1:1, 共10例有吸烟史。从诊断为NSCLC至发生LM, 时间为8个月-63个月, 中位时间28.5个月; 从开始接受TKIs治疗到诊断LM的时间为5个月-34个月, 中位时间为14个月; 诊断LM时患者的美国东部肿瘤协作组(Eastern Cooperative Oncology Group, ECOG)评分为1分-3分。18例患者中, 3例(16.67%)脑脊液细胞学阴性, 3例(16.67%)头颅MRI阴性, 18例(100.00%)脑脊液ctDNA的NGS检测均阳性。所有患者均接受过TKIs的治疗, 6例(33.33%)合并脑实质转移(brain metastasis, BM)的患者中, 4例接受了全脑放疗(whole brain radiation therapy, WBRT)。相关内容详见表 1。

**1:** LM患者的基本情况 Characteristics of patients with LM

No.	Sex	Age at LM	Histology	Initial stage	Smoking	Median time from cancer diagnosis to LM (mon)	Time from the start of TKI to LM (mon)	GCOG at LM	CSF cytologic testing	Brain and spinal MRI	CSF NGS	BM	Baseline mutation	TKI before LM	TKI after LM	WBRT
1	F	62	AD	IV	No	25	25	2	P	P	P	N	*EGFR*	Icotinib	Osimertinib	N
2	M	46	AD	II	Yes	63	10	1	P	N	P	N	*EGFR*	Gefitinib	Osimertinib	N
3	M	50	AD	II	Yes	31	6	2	N	P	P	N	*EGFR*	Icotinib	Osimertinib	N
4	F	38	AD	IV	No	13	7	2	P	N	P	N	*EGFR*	Gefitinib	Osimertinib	N
5	M	56	AD	IV	Yes	35	34	1	N	P	P	Y	*EGFR*	Gefitinib, Erlotinib	Osimertinib	N
6	F	58	AD	IV	No	29	28	2	P	P	P	N	*EGFR*	Gefitinib	Osimertinib	N
7	M	59	AD	III	Yes	28	23	3	P	P	P	N	*EGFR*	Osimertinib	Gefitinib	N
8	F	51	AD	IV	Yes	30	30	2	P	P	P	N	*EGFR*	Gefitinib, Crizotinib	Osimertinib	N
9	F	39	AD	III	No	36	21	2	P	P	P	N	*EGFR*	Icotinib	Erlotinib, Osimertinib	N
10	M	54	AD	III	Yes	31	23	1	P	P	P	Y	*EGFR*	Icotinib, Erlotinib	Osimertinib	Y
11	M	48	AD	III	Yes	12	9	2	P	P	P	Y	*EGFR*	Gefitinib, Erlotinib	Osimertinib, Crizotinib	Y
12	F	62	AD	IV	No	8	8	2	P	P	P	Y	*ALK*	Crizotinib, Brigatinib	Alectinib, Lorlatinib	Y
13	F	44	AD	IV	No	11	10	1	P	P	P	Y	*ALK*	Crizotinib, Alectinib	Lorlatinib	N
14	F	48	AD	IV	No	29	17	2	P	N	P	N	*ALK*	Lorlatinib	Alectinib	N
15	M	33	AD	IV	Yes	10	9	1	P	P	P	N	*ALK*	Crizotinib	Lorlatinib	N
16	F	33	AD	III	No	15	11	2	N	P	P	N	*ALK*	Crizotinib	Alectinib	N
17	M	46	AD	III	Yes	9	5	3	P	P	P	Y	*ALK*	Crizotinib, Brigatinib	Lorlatinib, Alectinib	Y
18	M	39	AD	IV	Yes	39	19	3	P	P	P	N	*ROS1*	Crizotinib Cabozantinib	Lorlatinib	N
LM: leptomeningeal metastases; ECOG: Eastern Clinical Oncology Group; MRI: magnetic resonance imaging; BM: brain metastases; TKI: tyrosine kinase inhibitor; WBRT: whole brain radiation therapy; F: female; M: male; AD: adenocarcinoma; N: negative; P: positive; EGFR: epidermal growth factor receptor; ALK: anaplastic lymphoma kinase; ROS1: ROS proto-oncogene 1, receptor tyrosine kinase.																

### 2.2 脑脊液及外周血中的基因突变情况

18例患者的详细基因突变情况见表 2, 其中*EGFR*、*ALK*、*ROS1*定义为基础突变。基础突变中11例(61.11%)为*EGFR*, 6例(33.33%)为*ALK*, 1例(5.56%)为*ROS1*。18例(100.00%)CSF样本的ctDNA均检测到靶向的基因突变, 而外周血中仅12例(66.67%)阳性, 差异有统计学意义(*P*=0.006)。此外, 基础突变基因的脑脊液丰度均明显高于血浆丰度(均值33.78% *vs* 1.58%), 且差异有明显统计学意义(*P* < 0.001)(图 2)。

**2:** 患者二代基因测序结果汇总 A summary of NGS results

No.	Gene	CSF (NGS)	Plasma (NGS)	Else (NGS)
1	*EGFR*	*EGFR* 19del (E746_A750del) (66.8%); *ATRX* MM (T429I) (34.8%); *MET* MM (I214M) (14.9%); *SETD2* MM (K2541N) (17.1%); *TP53* MM (R273H) (2.7%); *TP53* MM (H179Q) (0.7%)	*EGFR* 19del (E746_A750del) (0.9%); *ATRX* MM (T429I) (2.0%); *SETD2* MM (K2541N) (1.4%); *TP53* MM (R273H) (2.6%); *TP53* MM (H179Q) (0.3%)	
2	*EGFR*	*EGFR* 19del (L747_A750del) (18.3%); *APC* FM (Q1062*) (12.6%); *SKP2* MM (R154W) (9.1%)	*EGFR* 19del (L747_A750del) (5.6%); *APC* FM (Q1062*) (10.1%); *LRP1B* MM (G2173E) (4.7%); *SKP2* MM (R154W) (5.7%)	Hydrothorax:*EGFR* 19del (L747_A750del) (80.9%); *APC* FM (Q1062*) (73.5%); *TERC* CNG (CN=3.5); *SRC* CNG (CN=2.0); *LRP1B* MM (G2173E) (0.6%); *SKP2* MM (R154W) (22.5%) Tissue:*EGFR* 19del (L747_A750del) (29.9%); *APC* FM (Q1062*) (33%); *SKP2* MM (R154W) (13.0%)
3	*EGFR*	*EGFR* L858R (76.9%); *CDK4* CNG (CN=4.5); *SMARCA4* TM (W922*) (42.5%); *MTOR* MM (M1650I) (18.3%)	*EGFR* L858R (0.2%)	
4	*EGFR*	*EGFR* 19del (E746_A750del) (37.2%); *LRP1B* MM (V1432E) (0.6%)	Negative	
5	*EGFR*	*EGFR* L858R (52.3%); *EGFR* T790M (0.4%); *EGFR* CNG (CN=2.3); *TP53* FM (I162SfsX5) (36.8%)	*EGFR* L858R (0.6%); *EGFR* T790M (0.1%); *TP53* FM (I162SfsX5) (0.1%)	
6	*EGFR*	*EGFR* 19del (K745_A750del) (54.8%); *TP53* mutation (G783-1T) (76.7%)	Negative	
7	*EGFR*	*EGFR* 19del (L747_A750del+T751del) (4.2%); *CTNNB1* MM (E77K) (5.5%)	Negative	
8	*EGFR*	*EGFR* (L858R) (83.5%); *EGFR* CNG (CN=2.0); *MCL1* CNG (CN=2.6); *TERC* CNG (CN=2.0); *TERT* CNG (CN=2.1); *MED12* mutation (G1249-1T) (9.0%); *RB1* TM (Q471X) (88.6%); *TP53* FM (L93fs) (88.3%)	*EGFR* (L858R) (3.5%); *EGFR* (T790M) (0.2%); *PIK3CA* mutation (E545K) (0.9%); *TP53* FM (L93fs) (1.4%)	
9	*EGFR*	*EGFR* (L858R) (0.6%); *TP53* (R273H) (1.7%)	*TP53* (R273H) (0.1%)	
10	*EGFR*	*EGFR* 19del (T751_I759del) (6.6%); *EGFR* (T790M) (2.1%); *AKT1* MM (M178V) (3.8%); *ATR* MM (Q1732L) (1.0%); *TP53* MM (C176F) (3.7%)	*EGFR* 19del (T751_I759del) (3.3%); *EGFR* (T790M) (0.1%); *AKT1* MM (M178V) (2.5%); *TP53* MM (C176F) (0.8%)	Tissue:*EGFR* 19del (T751_I759del) (43.0%); *EGFR* (T790M) (0.3%); *AKT1* MM (M178V) (29.0%); *TP53* MM (C176F) (58.0%)
11	*EGFR*	*EGFR* (L858R) (1.58%); *EGFR* CNG (CN=36.8); *TP53* MM (A282G) (97.64%); *MYC* CNG (CN=53.9); *RB1* CND (CN=0.0)	*EGFR* (L858R) (1.29%); *TP53* MM (A282G) (1.03%); EGFR CNG (CN=3.3); *ERBB2* CNG (CN=2.4); *MYC* CNG (CN=3.4)	
12	*ALK*	*EML4-ALK* (E6:E20) (1.6%)	Negative	
13	*ALK*	*EML4-ALK* (E18:E20) (54.9%); *MET* CNG (CN=5.2)	*EML4-ALK* (E18:E20) (8.7%); *MET* MM (L1195V) (4.1%); *MET* MM (D1228N) (2.8%)	
14	*ALK*	*EML4-ALK* (E13:E20) (0.5%); *CDKN2A* (c.151-557_180del) (2.0%); *PDCD1* (c.711_*384del) (2.4%)	*EML4-ALK* (E13:E20) (0.1%); *CDKN2A* (c.151-557_180del) (0.03%)	
15	*ALK*	*EML4-ALK* (E13:E20) (16.6%)	*EML4-ALK* (E13:E20) (0.7%)	
16	*ALK*	*CNTNAP5-ALK* (C2:A20) (63.1%); *WDR43-ALK* (Wintergenic: A20) (67.7%); *MET* MM (P1330L) (50.42%)	*Negative*	
17	*ALK*	*EML4-ALK* (E13:E20) (29.7%); *MET* CNG (CN=10.3); *MET* MM (G667C) (0.2%); *ABL1* MM (A945T) (0.2%); *AR* MM (A897T) (0.8%); *CHEK2* MM (K373E) (2.6%); *ESR1* MM (R394C) (0.6%); *GRIN2A* MM (D45N) (0.2%); *JAK3* MM (S931L) (0.6%); *KEAP1* MM (L81Q) (0.6%); *LRP1B* MM (P1139H) (1.5%); *MYCN* MM (T8I) (0.6%); *NTRK1* MM (E295G) (1.3%); *NTRK1* MM (P302S) (1.4%); *NTRK3* MM (R138Q) (0.3%); *SETD2* MM (H2409L) (1.0%); *SOX2* MM (R57H) (0.2%); *SOX2* MM (L127Q) (0.9%); *VEGFA* MM (R396S) (0.5%)	Negative	
18	*ROS1*	*CD74-ROS1* (E6:E34) (38.8%); *ROS1* (G2032R) (39.8%); *NF1* (A702H) (26.3%)	*CD74-ROS1* (E6:E34) (0.3%)	
CN: copy number; CNG: copy number gain; CND: copy number decrease; MM: missense mutation; DM: deletion mutation; FM: frameshift mutation; TM: truncated mutation; TP53: tumor protein *tp53* gene; MET: mesenchymal-epithelial transition factor; ERBB2: human epidermal growth factor receptor 2; RB1: retinoblastoma 1 gene; CDK4: cyclin-dependent kinase 4 gene; APC: WNT signaling pathway regulator gene; NF1: neurofibromin 1; LRP1B: LDL receptor related protein 1B; SMARCA4: SWI/SNF related, matrix associated, actin dependent regulator of chromatin, subfamily a, member 4; MTOR: mechanistic target of rapamycin; MCL1: MCL1 apoptosis regulator; TERC: telomerase RNA component; TERT: telomerase reverse transcriptase; MED12: mediator complex subunit 12; PIK3CA: phosphatidylinositol-4, 5-bisphosphate 3-kinase catalytic subunit alpha; MYC: v-myc avian myelocytomatosis viral oncogene homolog gene; PDCD1: programmed cell death 1; WDR43: WD repeat domain 43; ABL1: ABL proto-oncogene 1; AR: androgen receptor; AKT1: AKT serine/threonine kinase 1; ATR: ATR serine/threonine kinase; ATRX: ATRX chromatin remodeler; CHEK2: checkpoint kinase 2; CDKN2A: cyclin dependent kinase inhibitor 2A gene; CNTNAP5: contactin associated protein family member 5; CTNNB1: catenin beta 1; ESR1: estrogen receptor 1; GRIN2A: glutamate ionotropic receptor NMDA type subunit 2A; JAK3: Janus kinase 3; KEAP1: kelch like ECH associated protein 1 gene; LRP1B: LDL receptor related protein 1B; MYCN: v-myc avian myelocytomatosis viral oncogene neuroblastoma derived homolog; NTRK1: neurotrophic receptor tyrosine kinase 1; NTRK3: neurotrophic receptor tyrosine kinase 3; SETD2: SET domain containing 2; SOX2: SRY-box transcription factor 2; VEGFA: vascular endothelial growth factor A; SETD2: SET domain containing 2; SKP2: S-phase kinase associated protein 2; SRC: SRC proto-oncogene.				

**2:**
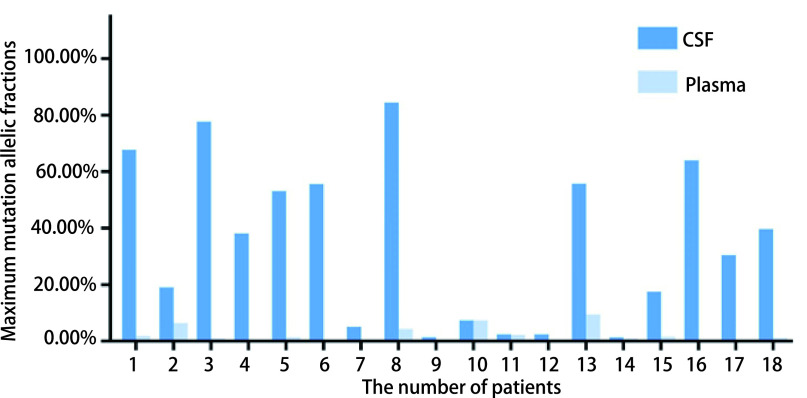
脑脊液及外周血中基础突变的脑脊液丰度和血浆丰度 Maximum mutation allelic fractions in CSF and plasma

脑脊液样本中检测出较多的单核苷酸变异(single-nucleotide variations, SNV)和拷贝数变异(copy number variants, CNV)(图 3), 数量均多于血液样本(SNV: 64 *vs* 28, CNV: 11 *vs* 3), 但差异无统计学意义(*P*=0.052, *P*=0.170), 且发现了较多的独有突变, 例如No.1患者中*MET*(I214M), No.3患者中*CDK4*、*SMARCA4*(W922*)、*MTOR*(M1650I), No.4患者中*LRP1B*(V1432E)等(见表 2)。此外, 在检测前使用多种靶向药物的患者中, 脑脊液和血液中SNV数目均多于仅接受过单一靶向药物治疗的患者(脑脊液：2.7 *vs* 1.4, 外周血：4.63 *vs* 1.75), 差异无统计学意义(*P*=0.304, *P*=0.961)。

**3:**
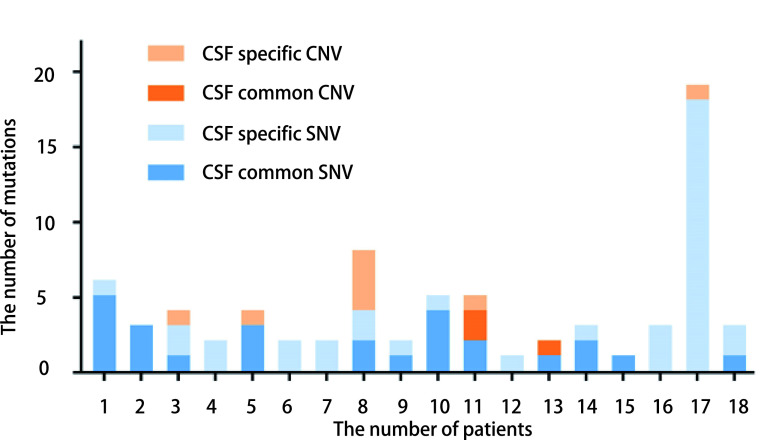
各个患者脑脊液样本中检测出的单核苷酸变异和拷贝数变异数目统计 The number of SNV and CNV mutations in CSF

11例*EGFR*突变的患者中, 6例(54.55%)为19del突变, 5例(45.45%)为L858R突变。伴随突变中, 脑脊液*TP53*检出率为63.64%(7/11), 高于血液中的检出率为54.55%(6/11);其他突变类型包括*EGFR* T790M(2/11, 18.18%)、*MET*(1/11, 9.09%)阳性率较低, 但这两种突变在有*TP53*突变的患者中检出率相对较高(42.86% *vs* 0.00%)。

在6例*ALK*突变的患者中, 5例均为*EML4-ALK*融合突变, 这其中E13:E20有3例, E6:E20和E18:E20各1例。另外1例(No.16)同时存在较为少见的*CNTNAP5-ALK*(C2:A20)和*WDR43-ALK*(Wintergenic: A20)融合突变。此外这些患者中*MET*突变检出率也相对较高(3/6, 50.00%)。

仅1例*ROS1*突变的患者, 为*CD74-ROS1*(E6:E34)融合突变, 在应用克唑替尼、卡博替尼、劳拉替尼靶向治疗后, 均快速的产生耐药, 考虑与出现继发性的耐药突变*ROS1*(G2032R)错义突变和*NF1*(A702H)移码突变相关, 而这两种突变在该患者的血液样本中均未能检测出来。

## 3 讨论

LM发生的中位时间约在TKIs开始使用后的14.2个月^[2]^, 目前该病的治疗仍较为棘手, 需要制定个体化的治疗方案, 其中首要的是明确基因突变的状态, 如存在*EGFR*突变, 可选择奥希替尼、阿法替尼、厄洛替尼等TKIs, 其中三代TKI奥希替尼因其良好的中枢穿透性和可靠的抗肿瘤效果^[8]^, 不管患者是否存在*EGFR* T790M突变, 均是推荐优先选择的药物^[9]^。约5%的NSCLC患者存在*ALK*融合基因, 治疗上可选择一代克唑替尼, 二代艾乐替尼、色瑞替尼及布加替尼或三代劳拉替尼, 针对合并LM的患者, 艾乐替尼由于拥有相对更好的中枢控制率因而作为优选药物^[3]^。当靶向药物耐药后, 全身治疗方面可尝试全身化疗、免疫治疗, 但由于血脑屏障的限制, 效果均不太理想; 局部治疗可尝试鞘内化疗及全脑放疗, 由于鞘内大剂量多次注射化疗药物会产生较重的神经系统毒性, 而全脑全脊髓放射治疗易伴发严重的骨髓抑制, 且易引发高病死率^[10]^, 因此两者的临床使用仍存在较大争议。

TKIs治疗一段时间后, 常伴发着无法避免的耐药问题, 其主要原因是*EGFR* T790M等各种获得性耐药突变的出现。值得注意的是, 多项研究^[11, 12]^表明颅内转移灶和颅外转移灶不同的基因突变情况。Nanjo等^[11]^研究发现在*EGFR*突变的LM患者中, 出现TKI耐药后重新进行基因检测, T790M在肺组织中的突变率明显高于CSF的突变率(55% *vs* 8%)。此外Ruppert等^[13]^研究证实NSCLC患者的颅内与颅外病灶对EGFR-TKI的药物敏感性也不同。因此耐药后再次明确中枢局部的基因突变状态显得尤为重要。

ctDNA是指来源于凋亡的肿瘤细胞或存活的肿瘤细胞分泌释放出的DNA, 其携带有原发灶的分子遗传学特点^[5, 14]^。相较于组织活检, ctDNA液体活检取材安全、便捷, 可以准确地检测出多种耐药基因。此外, 靶向治疗前后的ctDNA突变谱组和突变频率的动态变化, 可用于实时监测药物的疗效, 如敏感基因突变丰度降低, 常提示较好的疗效及预后; 如丰度无明显变化或升高, 常预示着耐药或复发^[15-17]^, 且相较于影像学的复发评估, 可提前约11个月, 敏感性可达93%^[18]^。

由于血脑屏障(blood brain barrier, BBB)的限制, LM的特征性分子学标志物难以进入血液循环, 且外周血的ctDNA检测结果中, 肿瘤原发灶、其他颅外转移灶产生的ctDNA会对其造成一定程度的干扰, 因此CSF样本比血液样本能更真实地反映LM突变情况^[19, 20]^。已有研究^[6, 7]^显示CSF中的ctDNA含量比血浆中的含量更高, 能更全面地表征颅内肿瘤的基因组改变。因此本研究利用NGS技术检测NSCLC患者CSF中的ctDNA, 并对比了同期的血液样本中的基因谱, 结果显示CSF样本的靶基因突变检出率和丰度均明显高于外周血, 虽然本研究样本量较小, 但是与既往的相关研究结果^[6, 19, 21, 22]^拥有一致的趋势, 进一步证实了CSF中ctDNA检测对于指导NSCLC伴LM患者治疗的重要价值。

在本研究所有*EGFR*突变的患者中, 发生TKI耐药后CSF中T790M突变率低于颅外病灶的突变率, 此结果与既往相关研究结果^[23, 24]^一致。这可能是由于BBB在中枢形成了一个肿瘤细胞的“避难所”, 限制了TKI的渗透, 因而药物导致的继发性T790M耐药突变相对较少。此外, 有报道^[25, 26]^证实在常规剂量的TKI耐药后, 增大剂量可有效控制颅内的病灶。综合以上的研究结论, 中枢性的耐药机制可能不同于其他病灶, 主要原因并不是耐药基因的产生, 而是药物无法在中枢达到有效的治疗浓度。及时有效的明确中枢病灶的基因突变情况, 可明确不同患者耐药的真实原因, 指导后续治疗是增大TKI剂量还是更换种类, 制定更为科学有效的治疗方案。

*TP53*突变也呈现较高的检出率, 多项研究^[27, 28]^证实其是NSCLC的独立预后因素, 常提示较短的无进展生存期(progression-free survival, PFS)及总生存期(overall survival, OS)。其中的机制尚无定论, Hou等^[29]^发现*TP53*可降低中国NSCLC患者对TKIs的反应性, 可能与*EGFR*-TKIs的原发耐药有关, 进而造成预后不良。本研究中*TP53*脑脊液中的突变率略高于血液, 因此推测其可能与LM的发生及耐药相关, 有待后续相关研究证实。

另外, 本研究中发现了3例患者存在罕见的非经典突变。No.10患者为*EGFR*第19外显子非移码缺失突变, 突变型为T751_I759del, 既往国内外文献中仅有过1例报道^[30]^。该患者的外周血及肺癌组织中检测出了相同的突变类型, 体现了CSF与其他组织类型的一致性。患者在多种TKIs及放疗、化疗的综合治疗下, OS达到51个月。No.16患者同时存在两种非经典*ALK*融合基因, *CNTNAP5-ALK*(C2:A20)和*WDR43-ALK*(Wintergenic:A20), 且在初诊及出现LM后复查均为相同的基因融合类型, 既往文献中没有报道过类似的病例, 虽经积极的化疗及靶向治疗, 但OS仅18个月, 考虑该两种融合基因可能与患者的耐药性相关, 相关可能的机制有待进一步的探索。No.18为*ROS1*突变的多发耐药患者, 是国内外报道的首个同时存在*CD74-ROS1*、*NF1*突变的病例, 研究^[31]^显示两种突变共同作用, 可促使RAS及下游通路激活, 参与肿瘤发生发展, 从而导致患者对于多种靶向药物的耐药。后2例患者的特有突变都仅在患者的CSF样本中检测出来, 进一步证实了脑脊液用于液体活检的全面性和优越性。

综上所述, 通过NGS液体活检技术, 检测LM患者CSF中的ctDNA变化, 相较于外周血, 可以更加准确、全面地反映出LM的真实基因突变状态, 在指导用药、监测耐药、疗效评估及预后判断等方面具有广泛的应用前景。本研究样本量有限, 后续将进一步扩大样本量验证相关结论, 并探索TKIs治疗前后的ctDNA动态变化, 以期为TKI耐药后的治疗提供依据, 推进更加精准的肿瘤个体化治疗。
